# Novel Synthesis of Cellulose-Based Diblock Copolymer of Poly(hydroxyethyl methacrylate) by Mechanochemical Reaction

**DOI:** 10.1155/2014/127506

**Published:** 2014-03-04

**Authors:** Takeshi Ohura, Yusaku Tsutaki, Masato Sakaguchi

**Affiliations:** ^1^Faculty of Agriculture, Meijo University, 1-501, Shiogamaguchi, Nagoya 468-8502, Japan; ^2^Institute for Environmental Sciences, University of Shizuoka, 52-1 Yada, Shizuoka 422-8526, Japan

## Abstract

The mechanical fracture of polymer produces polymeric free radical chain-ends, by which liner block copolymers have been synthesized. A diblock copolymer of microcrystalline cellulose (MCC) and poly 2-hydroxyethyl methacrylate (pHEMA) was produced by the mechanochemical polymerization under vacuum and room temperature. The fraction of pHEMA in MCC-block-pHEMA produced by the mechanochemical polymerization increased up to 21 mol% with increasing fracture time (~6 h). Then, the tacticities of HEMA sequences in MCC-block-pHEMA varied according to the reaction time. In the process of mechanochemical polymerization, cellulose could play the role of a radical polymerization initiator capable of controlling stereoregularity.

## 1. Introduction

Cellulose is the most abundant biological resource on the earth, which has been applied to composite materials, textile, drug delivery systems, personal care products, and so forth [1]. Therefore, making efficient use of cellulose would show superiority compared to other synthetic polymers in terms of ecological and environmental properties. The glycosylation of cellulose has been well studied for a long time and all over the world, by which energy and food problems are hoped to be solved. However, cellulose is structured by robust *β*-1,4-glycosidic linkages, so that the effectively and safety techniques have not been established. On the other hand, chemical modification techniques of cellulose have been developed to provide further functions. For instance, celluloid (cellulose nitrate) is the earliest modified cellulose, which had thermoplasticity as additional function. The recent advances of cellulose modifications are expanding from esterification of cellulose (e.g., cellulose nitrate and cellulose acetate) to graft copolymerization of cellulose [1]. Note that almost all of those modified cellulose are graft polymer using hydroxyl group of cellulose.

The mechanochemical reaction has been known to be classical procedure for synthesizing linear block copolymer [2]. The reaction processes are initially triggered by end-chain radicals (called mechanoradicals) produced by mechanical fracture of polymer, and the mechanism is provided in Figure 4.

Indeed, the mechanochemical reaction was applied to synthesize block and graft copolymers of cellulose and vinyl monomers, resulted in the block copolymers being produced by the mechanical fracture [2]. Furthermore, such mechanoradicals provide us information of molecular motion of chain by using electron spin resonance (ESR) [3–6].

Sakaguchi et al. have revealed the scission of the *β*-1,4-glycosidic linkages of bacterial cellulose (BC) fractured mechanically under vacuum at 77 K from the ESR spectra [7, 8]. In addition, diblock copolymer of BC and poly(methyl methacrylate) was produced by the mechanical fracture, indicating that mechanoradicals behave like radical polymerization initiator. The greatest benefit of mechanoradicals will be a polymerization procedure with ultimate low environmental burden. However, the potentiality of mechanoradical polymerization including the adaptivity and versatility has been unsure at present, so that further investigations are needed to reveal the characterization. In the current study, we synthesize novel diblock copolymer of accessible and low-cost cellulose, MCC, and biocompatible materials, HEMA, by mechanical fracture at room temperature and report the function of MCC on the polymerization.

## 2. Experimental

### 2.1. Synthesis of MCC-Block-pHEMA

The diblock copolymers of MCC and pHEMA (MCC-block-pHEMA) were produced as follows: HEMA (50 mg, Wako Pure Chemicals, Osaka, Japan) purified by freeze-pump-thaw method was introduced into handmade glass vessel (50 mL) containing prevacuum-dried MMC (50 mg, Aldrich, USA) and alumina ball (10 g, 2 mm in diameter), and the glass vessel was sealed off from the vacuum line, set to shaker (shaking speed: 300 rpm, amplitude: 40 mm, SA-300, Yamato Scientific Co., Ltd. Tokyo, Japan) and milled in vacuum at room temperature for predetermined time (~6 h). After that, all products were sequentially washed by Soxhlet extraction with methanol for 16 h to remove unreacted HEMA, and the resultant residue was dried in oven at 80°C for 12 h.

### 2.2. Acetylation

The acetylation of MCC and MCC-block-pHEMA synthesized was performed to be analyzed by ^1^H-NMR. A mixture of acetic acid (0.57 mol) and trifluoroacetic acid anhydride (0.436 mol) was held at 50°C for 20 min. Then, MCC or MCC-block-pHEMA was introduced and the solution was acetylated at 50°C for 12 h. The acetylated product was precipitated with methanol, followed by filtration. After that, the precipitate was washed by methanol again and was dried in oven at 80°C for 12 h.

### 2.3. Analyses

The chemical structures of MCC and MCC-block-pHEMA produced were analyzed by FT-IR and ^1^H-NMR. FT-IR spectra were observed on a FT-IR 8400 (Shimadzu, Kyoto, Japan) using solid KBr pellets. ^1^H-NMR spectra were recorded on a JNM-BEX 270 NMR spectrometer at 270 MHz (JEOL, Tokyo, Japan) in CDCl_3_. TMS was used as the internal reference. The number and weight average molecular weights (*M*_*n*_ and *M*_*w*_) of samples were estimated by using GPC system (Waters 600E) with column Shodex GPC K-805L in chloroform at 40°C and 0.8 mL/min of flow rate. A calibration curve was obtained using polystyrene standards.

## 3. Results and Discussion

**Figure 1:**
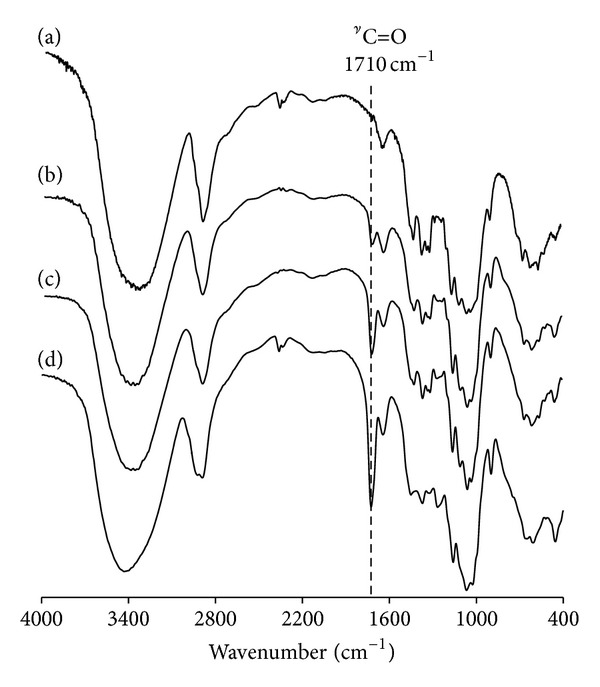
FT-IR spectra of the MCC-block-pHEMA produced by mechanochemical polymerization for (a) 0 h, (b) 1 h, (c) 3 h, and (d) 6 h.

**Figure 2:**
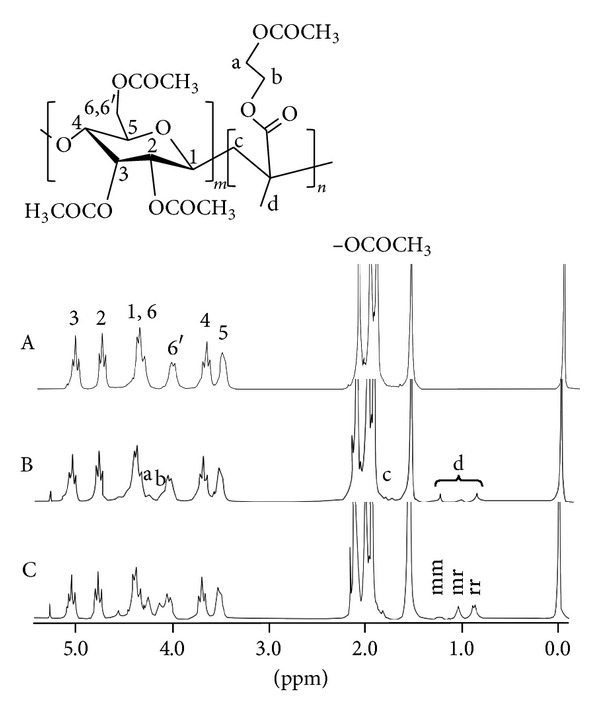
^1^H-NMR spectra of the acetylated MCC (A) as control and acetylated MCC-block-pHEMA produced by mechanochemical polymerization for (B) 1 h and (C) 6 h.

**Figure 3:**
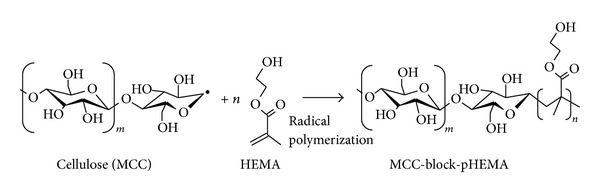
Reaction scheme of MCC-block-pHEMA production initiated by MCC mechanoradical and sequentially reacted with HEMA.

**Figure 4:**
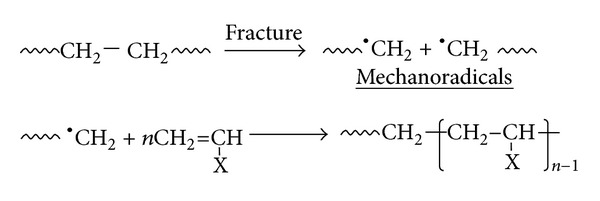


It has been widely investigated that mechanical destruction of cellulose results in the scission of the polymer main-chain, followed by the production of radicals even under room temperature [9–11]. This fact implies that cellulose fractured mechanically is possible to react as radical initiator. Here, we performed the production of novel diblock copolymer of cellulose (MCC) and pHEMA using mechanochemical polymerization.  Figure 1 shows the FT-IR spectra of MCC-block-pHEMA obtained by each reaction time of the mechanochemical polymerization. Peak at 1,710 cm^−1^ for C=O group of pHEMA was observed in all reactants, but not for samples for 0 h and without alumina ball. Sakaguchi and Sohma indicated that material with low molecular weights (~100) has no function in mechanochemical polymerization [12], so that current polymerization will be attributed to the scission of MCC, but not to self-polymerization of HEMA. In addition, the intensity of peaks obviously increased with an increase of reaction time from 1 h to 6 h (see Figure 2). Indeed, the molar ratios of pHEMA in MCC-block-pHEMA calculated by ^1^H-NMR increased from 7 mol% for 1 h to 37 mol% for 6 h (Figure 3), and those degrees of polymerization (DP) of pHEMA calculated from corresponding *M*_*n*_ values of the copolymer chains also increased from 23 to 69, respectively (Table 1). These facts indicate that radical polymerization of HEMA proceeded successfully from chain-end-type radicals of MCC cleaved by mechanically fracture. The putative reaction scheme is shown in Figure 3. On the other hand, *M*_*n*_ and *M*_*w*_/*M*_*n*_ of the MCC-block-pHEMA were somewhat consistent regardless of the reaction time (Table 1). It is suggested that polymerization of HEMA and mechanical destruction of solid MCC could also occur simultaneously in the process.

As one of function of cellulose posing, it has been known to be chiral recognition, that is, act as chiral adsorbent [13]. So, we next investigated the tacticity of pHEMA in the MCC-block-pHEMA produced by the mechanochemical reaction using ^1^H-NMR (Figure 3). At earlier stage (1 h) of the reaction, molar ratios of isotactic (mm), atactic (mr), and syndiotactic (rr) pHEMA were 39, 13, and 48 mol%, respectively (Table 1). For 3-hour reaction, those characteristics of tacticity were somewhat consistent with copolymer obtained for 1 h. At the latter stage (6 h), however, molar ratio of the isotactic unit decreased to 15 mol%, whereas molar ratio of the atactic unit increased to 38 mol%, and constant for syndiotactic fraction (Table 1). These results suggest that radical chain-end of cellulose surface bared by mechanochemical reaction has an ability of stereoregularity in the initial stage of radical polymerization; therefore, the ability could decay with progression of polymerization.

In this manner, the mechanochemical reaction indicates the possible presence of ecofriendly, simple, and functional polymerization to future generations. The MCC-block-pHEMA will be expected to application of the fields of not only ecologically friendly products (e.g., dispersant, builder, etc.) but also biocompatible materials. Further investigations will be needed to reveal the potential.

## Figures and Tables

**Table 1:** Characteristics of MCC-block-pHEMA produced by mechanochemical polymerization.

Run	Time (h)	pHEMA (mol%)	pHEMA DP^a^	Molecular weight		Tacticity (%)^b^		
				*M_n_* (×10^3^)	*M_w_/M_n_*	mm	mr	rr
1	0	—	—	46	1.5	—	—	—
2	1	7	23	54	1.4	39.1	13.1	47.8
3	3	30	52	30	1.6	41.1	17.1	41.8
4	6	37	69	32	1.6	14.5	38.2	47.3

^a^DP: degree of polymerization.

^b^mm: isotactic; rm: atactic; rr: syndiotactic.
